# 4-Hydroxy-2-nonenal Induces Apoptosis by Inhibiting AKT Signaling in Human Osteosarcoma Cells

**DOI:** 10.1155/2014/873525

**Published:** 2014-02-23

**Authors:** Guang-rong Ji, Nai-chun Yu, Xiang Xue, Zong-guang Li

**Affiliations:** Department of Orthopaedics, The 2nd Affiliated Hospital of Harbin Medical University, Harbin 150001, China

## Abstract

The onset of lipid peroxidation within cellular membranes is associated with changes in their physiochemical properties and enzymatic dysfunction of the membrane environment. There are increasing bodies of evidence indicating that aldehydic molecules generated endogenously during the process of lipid peroxidation are causally involved in most of the pathophysiological effects associated with oxidative stress in cells and tissues. 4-Hydroxy-2-nonenal (4-HNE), among them, is believed to be largely responsible for cytopathological effects observed during oxidative stress *in vivo* and has achieved the status of one of the best recognized and most studied of the cytotoxic products of lipid peroxidation. Here, we reported that 4-HNE treatment may induce cell death in MG63 human osteosarcoma cells. The 4-HNE treatment could activate caspase-3 and alter the Bax/Bcl-2 apoptotic signaling. All these changes are due to the inhibition of AKT activity by 4-HNE treatment, and we also found that the p70S6K activity, downstream factors of AKT, was also blocked by 4-HNE. Our results revealed the molecular mechanism of how 4-HNE induces cell death in MG63 human osteosarcoma cells, which contributes to the clinical treatment of cancer therapy.

## 1. Introduction

Reactive oxygen species (ROS) are formed in tissues as by-products of normal oxidation reactions and can be induced by environmental agents (e.g., ozone) and toxins (e.g., paraquat). They are capable of damaging biochemical compounds such as DNAs, proteins, and lipids and have been linked to many common human diseases such as cancers, heart attacks, stroke, and emphysema [1, 2]. Oxidative stress is known to induce apoptosis in a wide variety of cultured cells and is believed to cause apoptosis in various pathological conditions such as AIDS and neurodegenerative diseases [3, 4]. It has been shown that cells sustain progressive lipid peroxidation following an apoptotic signal, and it has been suggested that oxidative stress is a common mediator of apoptosis [5–7]. However, the essential biochemical events of apoptotic process in oxidative stress remain to be eliminated. Identification of the key mediator(s) for oxidative apoptosis will contribute to understanding the mechanism.

Oxidative free radicals are known to cause peroxidation of membrane polyunsaturated fatty acids. 4-Hydroxy-2-nonenal (4-HNE), an aldehyde product of membrane lipid peroxidation, can be produced by oxidative stimuli and has been detected in several diseases such as atherosclerosis, diabetes, and Parkinson's disease. The formation of 4-HNE and 4-HNE-protein conjugation has become a marker of oxidative stress in tissues or cells [8, 9]. Oxidative stress-induced apoptotic cell death is believed to be involved in the pathological generation of those oxidative stress-related diseases [6, 7]. Therefore, 4-HNE may be an important mediator of oxidative stress-induced apoptosis. It has been reported that 4-HNE and 4-HNE-protein adduct accumulate in neurons by oxidative insults and in lung cells by ozone exposure and thus are associated with the apoptotic events in these cells [10, 11]. Exogenously administrated 4-HNE has also been observed to form 4-HNE-protein adduct and induce apoptotic cell death in macrophages and neurons [12]. Much attention has recently been paid to 4-HNE-induced apoptotic cell death in the pathological development of neural and vascular degenerations, particularly because 4-HNE is not only a mediator for amyloid *β*-peptide-induced damage but also one effective component of oxidized low density lipoproteins [13, 14]. Although studies have shown that 4-HNE-mediated apoptotic cell death involves intracellular calcium uptake and 4-HNE directly interacts and activates c-Jun amino-terminal kinase (JNKs) [15], the mechanism of 4-HNE-induced apoptosis has not been clearly delineated.

Oxidative free radical-induced lipid peroxidation causes the formation of reactive aldehydes. These aldehydes have longer biological half-lives than free radicals and can diffuse from their site of formation to reach distant targets and therefore cause cellular damage [1, 2]. It has been suggested that these aldehydes may act as second toxic messengers of lipid peroxidation [16]. 4-Hydroxynonenal, or 4-hydroxy-2-nonenal (C9H16O2), is an *α*, *β*-unsaturated hydroxyalkenal that is produced by lipid peroxidation in cells. 4-HNE is the primary alpha, beta-unsaturated hydroxyalkenal formed in this process. 4-HNE has three reactive groups: an aldehyde, a double-bond at carbon 2, and a hydroxy group at carbon 4. It is found throughout animal tissues and in higher quantities during oxidative stress for the increase in the lipid peroxidation chain reaction and for the increase in stress events [17, 18]. Here, we reported that 4-HNE treatment may induce cell death in MG63 human osteosarcoma cells. The 4-HNE treatment may activate caspase-3 and alter the Bax/Bcl-2 apoptotic signaling. All these changes are due to the inhibition of protein kinase B (AKT) activity by 4-HNE treatment. We also found that the p70S6K activity, downstream factors of AKT, was blocked by 4-HNE. The present study reveals the molecular mechanism of how 4-HNE induces cell death in MG63 human osteosarcoma cells and thus benefits the clinical treatment of cancer therapy.

## 2. Materials and Methods

### 2.1. Reagents and Antibodies

The 4-hydroxynonenal was obtained from Biomol (Plymouth Meeting, PA, USA). Dulbecco's Modified Essential Medium (DMEM) and Fetal Bovine Serum (FBS) were purchased from GIBCO Invitrogen (Carlsbad, CA, USA). Anti-pAKT, anti-AKT, anti-cleaved-caspase-3, anti-p70S6K, and anti-p70S6K were purchased from Cell Signaling Technology (Danvers, MA, USA). Anti-Bcl2, anti-GAPDH, and anti-Bax antibodies were from Millipore (Billerica, MA, USA). Other reagents were of the highest purity available.

### 2.2. Cell Culture

A widely used human osteosarcoma cell line MG63 was chosen as *in vitro* experiment system. The cells were obtained from Shanghai Institute of Cell Biology (introduced from American Type Culture Collection). MG63 cells were derived from an osteosarcoma and widely used to study of the amplification process in tumors. The MG63 cells were plated in 6-well plates at 1.0 × 10^6^ cells/mL. The cells were incubated in DMEM containing 10% FBS plus antibiotics for 24 h in 5% CO_2_ at 37°C.

### 2.3. Pharmacological Manipulations

For oxidative stress induction in MG63 cells, we applied 4-HNE to these cells at the final concentration from 1 to 50 *μ*M for 4 h, and cells were harvested for subsequent examinations. Again, to study the time-dependent effect of 4-HNE on MG63 cells, cells were treated with 4-HNE for different times, from 0.5 h to 4 h, with the final concentration of 50 *μ*M. Cells with no additive were used as internal controls.

### 2.4. Western Blot

Western blot was performed as standard procedures. To extract proteins, cultured MG63 cells were sonicated with lysis buffer (PBS with 1% Triton X-100 and protease inhibitors). The cell lysate supernatants were harvested by centrifugation at 10 000 rpm for 10 min at 4°C. Protein concentrations of the cell supernatants were evaluated and measured by BCA Protein Assay kit (Thermo Fisher Scientific Inc., Rockford, IL, USA). Equal amount of the proteins from each extract were separated in a SDS-polyacrylamide gel (10%) with 5% stacking gel in SDS-Tris-glycine running buffer. The proteins were transferred electrophoretically using a PVDF membrane by standard procedures. The membranes were then blocked by 5% nonfat dry milk in PBST (PBS with 0.1% Tween-20, pH 7.6) for 1 h at room temperature and probed overnight by proper primary antibodies diluted in PBST at 4°C. The membranes were rinsed 3 times with PBST and incubated with proper secondary antibodies diluted in PBST for 1 h at room temperature. Then, the membranes were rinsed another 3 times with PBST at room temperature for 10 min, and proteins were detected by Super Signal enhanced chemiluminescence development (ECL) (Thermo Scientific Pierce) reagent and exposed to film (Kodak). The protein level quantification was carried out by ImageJ.

### 2.5. Statistical Methodology

All statistical analysis was performed by ImageJ software. Quantitative data were showed in $\overset{-}{x}\pm s$ using *t*-tests for comparisons. The values 0.05 (*), 0.01 (**), and 0.001 (***) were assumed as the level of significance for the statistic tests carried out.

## 3. Results

### 3.1. 4-HNE Induces Cell Death in MG63 Human Osteosarcoma Cells

**Figure 1:**
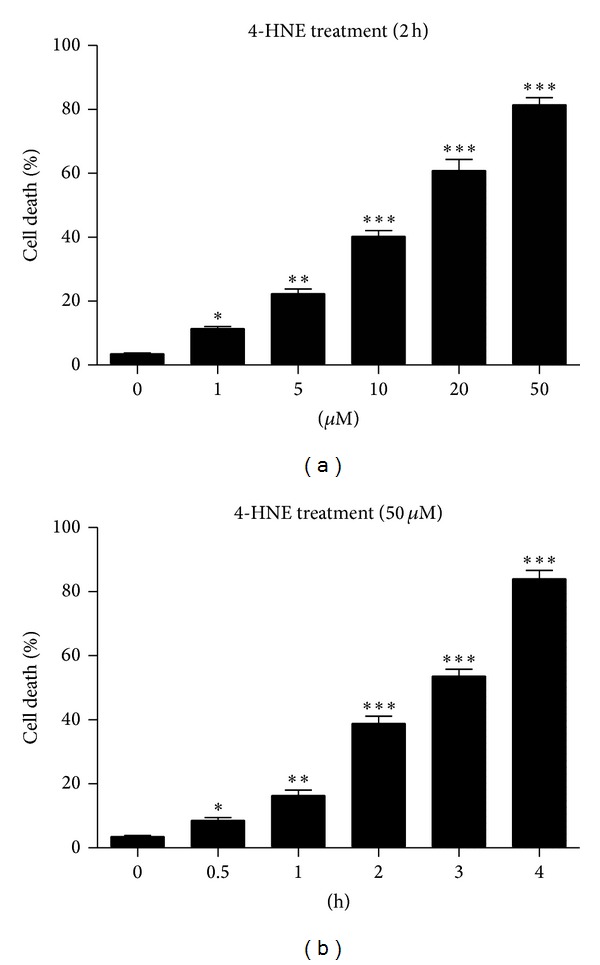
4-HNE induces cell apoptosis in MG63 cells. (a) Histograms showing the quantification of the cell death (%) in MG63 cells after 4-HNE treatment for 2 h and recovery for another 2 h. The final concentration of 4-HNE ranges from 1 to 50 *μ*M. Results are averages of three independent experiments. (b) Histograms showing the quantification of the cell death (%) in MG63 cells after 50 *μ*M 4-HNE treatment for different time points, from 0.5 to 4 h. Results are averages of three independent experiments.

4-Hydroxynonenal (4-HNE) is the product of membrane lipid peroxidation and reported as the mediator of oxidative stress induced cell apoptosis (Figure 1(a)). In order to investigate the effects of ROS on human osteosarcoma cell line MG63, we treated MG63 cells with 4-HNE (1 to 50 *μ*M) for 4 h. The subsequent Hoechst staining was carried out on the 4-HNE treated cells. Increased apoptotic cells were observed significantly as 4-HNE concentration improved correspondingly (*P* < 0.01 or *P* < 0.001) (Figure 1(b)). It is noted that nearly 80% of cells were apoptotic in 50 *μ*M 4-HNE treatment group, suggesting that the final concentration of 50 *μ*M was a good condition for apoptosis inducing in MG63 cells. To further study the time effect of 4-HNE on cell apoptosis, we applied 50 *μ*M 4-HNE to MG63 cells for different times. As shown, we found that the cell apoptosis aggravated as the time lapsed with significant statistic difference. These results gave the direct evidence showing that 4-HNE could induce cell death in MG63 human osteosarcoma cells.

### 3.2. 4-HNE Induces Caspase-3 Activation in MG63 Human Osteosarcoma Cells

**Figure 2:**
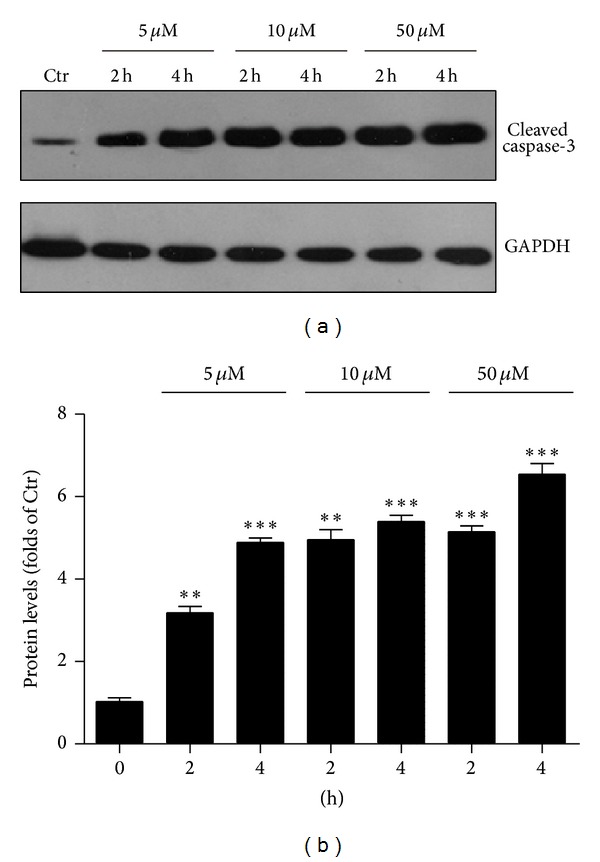
4-HNE activates caspase-3 in MG63 cells. Western blots (a) and histograms (b) showing the increasing of protein levels of cleaved caspase-3 by 4-HNE treatment (5 to 50 *μ*M, and 2 h to 4 h) in MG63 cells. Results are averages of three independent experiments.

To confirm the Hoechst staining results, we further evaluated biochemical effects of 4-HNE treated cells. The caspase-3 is cleaved at Asp-28-Ser-29 to generate the mature 17 kD fragment, which may cleave and activate caspase-6, -7, and -9, leading to apoptosis [19]. We found the cleaved caspase-3 level increased significantly (*P* < 0.001) after 4-HNE treatment, which showed that the apoptotic signaling was opened (Figures 2(a) and 2(b)). Thus, the present results clearly showed that the 4-HNE may induce cell death and activate caspase-3 cascades in human osteosarcoma cell line MG63.

### 3.3. 4-HNE Alters Bax/BcL-2 Ratio in MG63 Human Osteosarcoma Cells

**Figure 3:**
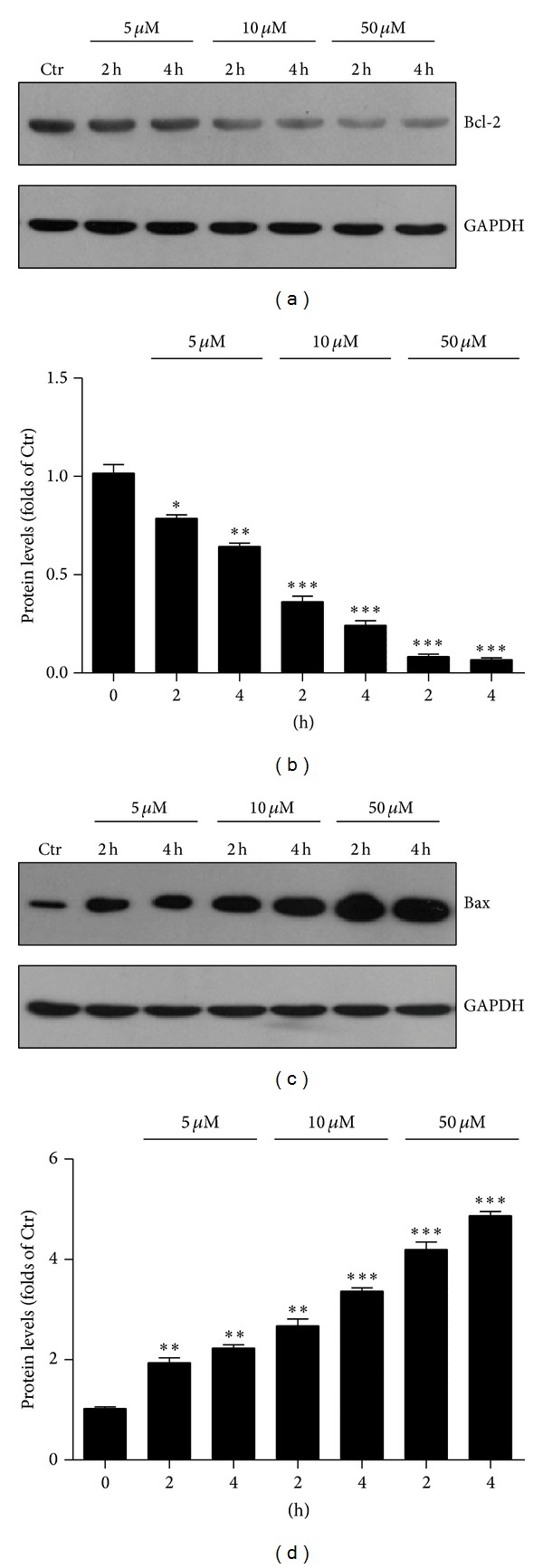
4-HNE activates apoptotic signaling in MG63 cells. (a)-(b) Western blots (a) and histograms (b) showing the decreasing of protein levels of BcL-2 by 4-HNE treatment (5 to 50 *μ*M, and 2 h to 4 h) in MG63 cells. Results are averages of three independent experiments. (c)-(d) Western blots (c) and histograms (d) showing the increasing of protein levels of Bax by 4-HNE treatment (5 to 50 *μ*M, and 2 h to 4 h) in MG63 cells. Results are averages of three independent experiments.

To study the effect of 4-HNE on the apoptosis of MG63 cells, we applied 4-HNE (5 to 50 *μ*M) on MG63 cells for 2 h and these cells were recovered for another 2 or 4 h. By biochemical analysis, we found that the Bcl-2 protein levels were reduced by 4-HNE treatment in a time- and dose-dependent manner (Figures 3(a) and 3(b)). On the other hand, the protein levels of Bax were constitutively increased by 4-HNE (Figures 3(c) and 3(d)). The reduced Bcl-2 and increased Bax by 4-HNE treatment would contribute to the activation of caspase-3 and finally the cell apoptosis. These results indicated that 4-HNE treatment altered the intracellular ratio of Bcl-2 and Bax, which may be the key events of the apoptosis in MG63 cells.

### 3.4. 4-HNE Inhibits AKT Activity in MG63 Human Osteosarcoma Cells

**Figure 4:**
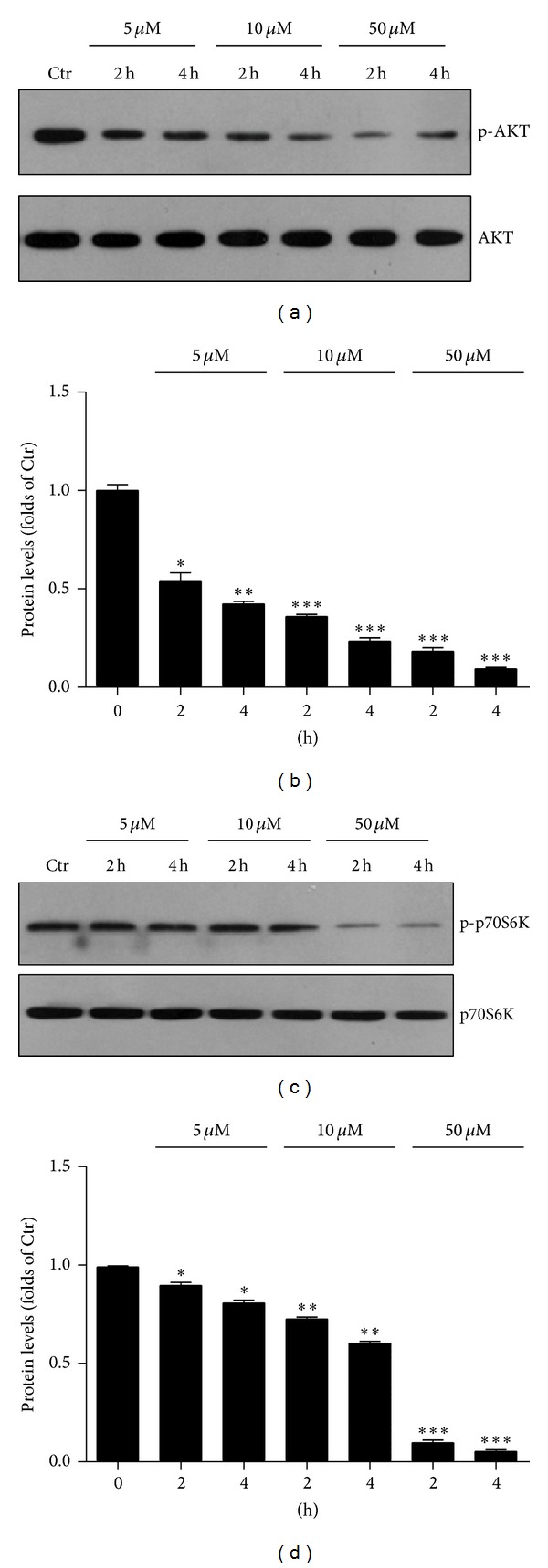
4-HNE inhibits AKT pathways in MG63 cells. (a)-(b) Western blots (a) and histograms (b) showing the decreasing of protein levels of pAKT by 4-HNE treatment (5 to 50 *μ*M, and 2 h to 4 h) in MG63 cells. Results are averages of three independent experiments. (c)-(d) Western blots (a) and histograms (b) showing the decreasing of protein levels of pp70S6K by 4-HNE treatment (5 to 50 *μ*M, and 2 h to 4 h) in MG63 cells. Results are averages of three independent experiments.

To study whether 4-HNE-induced cell death in MG63 cells was caused by AKT alterations, we examined the AKT phosphorylations in cells treated with 4-HNE. We found that the protein level of AKT-p (Thr 308) significantly decreased in 4-HNE treated MG63 cells in a time- and dose-dependent manner (Figures 4(a) and 4(b)). As control, the total level of AKT was not altered. Mammalian target of rapamycin (mTOR), downstream of AKT, is responsible for the protein synthesis, cell proliferation, cell size, and growth. The mTOR signaling could also prevent cell apoptosis in response to nutrient availability, oxygen, and energy levels [20, 21]. We wonder whether 4-HNE would reduce the activity of mTOR signaling, which contributes to cell apoptosis in MG63 cells. Our biochemical data showed the protein level of p70S6K (Thr 389), which was well-known markers of mTOR signaling, reduced after 4-HNE treatment (Figures 4(c) and 4(d)). The inhibition of mTOR signaling attenuated the cellular protection system and contributed to cell apoptosis in 4-HNE treated human osteosarcoma cell line MG63. All these results suggested that extrinsic 4-HNE may inactivate AKT/mTOR signaling and promote the cell apoptosis in human osteosarcoma cell line MG63.

## 4. Discussion

**Figure 5:**
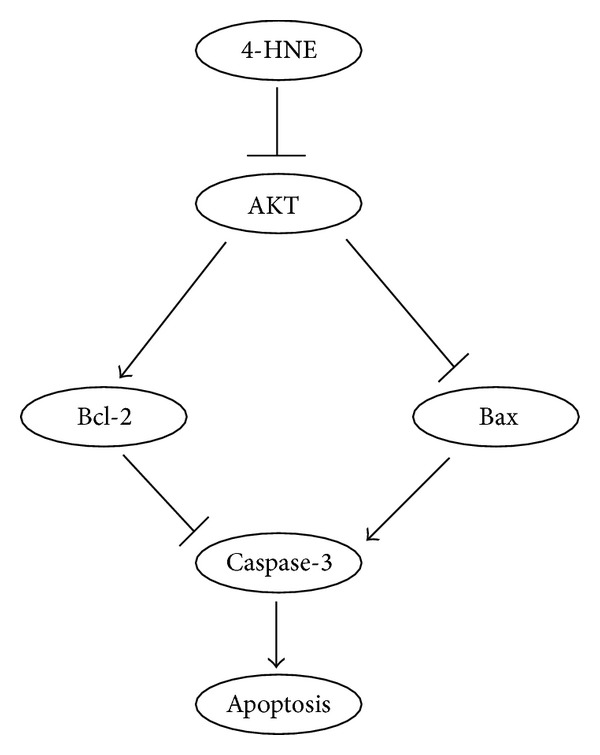
Models. Simplified scheme showed that 4-HNE could inactivate AKT activity, alter the ratio of BcL-2/Bax, induce caspase-3 cascades, and finally lead to apoptosis in MG63 cells.

In this study, we demonstrate that 4-HNE could induce dramatic apoptosis in MG63 human osteosarcoma cell line. 4-HNE treatment could alter the Bcl-2/Bax ratios and activate caspase-3 apoptotic cascades, which may be mediated by the inhibition of AKT pathways. Our work reveals the molecular mechanism of 4-HNE on the cell death of MG63 cells and contributes to the clinical cancer therapy (Figure 5).

The activation of apoptosis is controlled at multiple checkpoints within the cell. Upstream, the proapoptotic Bax and antiapoptotic Bcl-2 are membrane-bound pore-forming proteins that interact through heterodimerization. Bcl-2 is recognized as a novel type of multidrug-resistant protein that protects tumor cells from the cytotoxic effects of virtually every anticancer drug currently available. Bcl-2 is the first proto-oncogene that was found to aggregate tumor growth by decreasing the rate of apoptosis rather than by accelerating cell division. A second regulator of cellular responsiveness is the proapoptotic molecule Bax. Whereas Bcl-2 overexpression has been shown to inhibit apoptosis, a predominance of Bax to Bcl-2 accelerates apoptosis upon apoptotic stimuli. Bcl-2 and Bax interactions have often been presented as a model where the cell's fate can be changed by changing the balance or ratio of Bax and Bcl-2 protein expression [22, 23]. In the present study, we demonstrate that products of lipid peroxidation, HNE, could induce cell death in MG63 human osteosarcoma cells by altering Bcl-2/Bax ratio and activating caspase-3 cascades. AKT, a serine-threonine kinase, can function to either reduce or prevent cellular destruction from oxidants and play a critical role in cell survival. For AKT to be fully activated, the phosphorylation of its Thr 308 site is necessary, and helps to cell survival [24, 25]. The present study suggested that HNE may inhibit the activity of AKT, which contribute to the opening of cell apoptotic signaling (Figure 5). The present results revealed novel mechanisms of how HNE induces cell death in MG63 cells and helped in the clinical therapy of cancers, such as osteosarcomas.

Role of 4-HNE in signaling process appears to be intriguing because its effect is concentration dependent. At increasing concentrations, 4-HNE is reported to cause cell cycle arrest, differentiation, and apoptosis, while at lower concentrations it has been shown to promote proliferation in at least some cell types. Therefore, the regulation of the intracellular concentrations of 4-HNE may be crucial for cell cycle signaling. 4-HNE concentrations in cells increase during oxidative stress, but limited information is available regarding the normal physiological levels of 4-HNE in cells. Nonetheless, 4-HNE in varying amounts is present in all the cell lines and tissues we have examined so far. Since the formation of 4-HNE results from lipid peroxidation, an uncontrolled process depending on the redox status of cells, the intracellular levels of 4-HNE should be controlled through its metabolism and subsequent transport of the metabolites [26, 27]. Caspase-3 is a caspase protein that interacts with caspase-8 and caspase-9. It is encoded by the CASP3 gene. CASP3 orthologs have been identified in numerous mammals for which complete genome data are available. Caspase-3 is activated in the apoptotic cell both by extrinsic (death ligand) and intrinsic (mitochondrial) pathways. The zymogen feature of caspase-3 is necessary because if unregulated, caspase activity would kill cells indiscriminately. As an executioner caspase, the caspase-3 zymogen has virtually no activity until it is cleaved by an initiator caspase after apoptotic signaling events have occurred [16]. One such signaling event is the introduction of Granzyme B, which can activate initiator caspases, into cells targeted for apoptosis by killer T cells. This extrinsic activation then triggers the hallmark caspase cascade characteristic of the apoptotic pathway, in which caspase-3 plays a dominant role. In the intrinsic activation, cytochrome c works in combination with caspase-9, apoptosis-activating factor 1 (APAF-1), and ATP to process procaspase-3 [28, 29]. It has recently been reported that arginine-glycine-aspartate (RGD) containing peptides directly induces autoprocessing and activation of caspase-3, suggesting that caspase-3 may also be activated without signals from cell surface death receptors or from mitochondria [30]. In this study, we did not obtain any clear evidence of direct binding of HNE with caspase-3 (data not shown). However, this does not rule out the possibility that intracellular HNE or HNE-triggered alternation of the intracellular redox has some direct impact on caspases activity through their self-processing, because common to the caspase family is the existence of a reactive cysteine in the active site, and this reactive cysteine is sensitive to the redox status of the cell.

## 5. Conclusion

In summary, our results indicated that 4-HNE may induce the apoptosis in MG63 human osteosarcoma cells by inhibiting the AKT pathways and activating apoptotic cascades. The present findings suggested that therapies that specifically rise the 4-HNE levels in tumor cells may prove useful for the treatment of clinical cancers.
